# Complete Genome Sequence of a New Mastrevirus, Chickpea Redleaf Virus 2, from Australia

**DOI:** 10.1128/MRA.00602-19

**Published:** 2019-09-05

**Authors:** Fiona F. Filardo, Murray Sharman

**Affiliations:** a Ecosciences Precinct, Department of Agriculture and Fisheries, Brisbane, Queensland, Australia; DOE Joint Genome Institute

## Abstract

We present here the complete genome sequence of a novel mastrevirus isolated from Cicer arietinum (chickpea) from Australia. We propose the name chickpea redleaf virus 2.

## ANNOUNCEMENT

The genus Mastrevirus (family *Geminiviridae*) contains species that infect monocotyledons or dicotyledons. They contain a single-stranded circular DNA genome and are transmitted by leafhoppers (order *Hemiptera*, family *Cicadellidae*). Previous studies suggest that Australia may be a center of origin for dicot-infecting mastreviruses (1–3).

During virus surveys of pulse crops in 2017 in northern New South Wales (NSW) and southern Queensland (QLD), Australia, a total of 254 chickpea samples were collected for analysis. To determine positive mastrevirus samples, tissue blot immunoassays (TBIAs) were carried out according to Makkouk and Comeau (4) and processed using a polyclonal antiserum to chickpea chlorotic dwarf virus (CpCDV), which reacts with a broad range of Mastrevirus members (5). DNA was extracted from 23 of the 71 virus-positive chickpea samples using a BioSprint 15 workstation with a BioSprint 15 plant DNA kit (Qiagen) per the manufacturer’s instructions.

To identify the species of *Mastrevirus*, a 371-bp PCR product was amplified using mastrevirus primers Mastre_F3 (AGGAGAGGCACGTTNAGTGAC) and Mastre_R2 (GTACMGGWAAGACMWSHTGGG) (modified from references 1 and 6) using previously described conditions and cycling parameters (1, 6), except with a 58°C annealing temperature. Products were directly Sanger sequenced by the Australian Genome Research Facility (AGRF) and analyzed with Geneious 10.0 (Biomatters). Sequence analysis of the PCR products showed that the majority of mastrevirus infections were caused by a strain of chickpea chlorosis virus (CpCV). BLAST (7) analysis indicated that 12 isolates matched CpCV-E with 97 to 99% nucleotide (nt) identity to the isolate with GenBank accession number JN989431, 6 matched CpCV-A with 96 to 99% nt identity to the isolate with GenBank accession number GU256530, 3 matched CpCV-B with 98% nt identity to the isolate with GenBank accession number GU256531, and 1 matched CpCV-F with 97% nt identity to the isolate with GenBank accession number KC172700. However, one isolate, 5495, from chickpea showing strong reddening symptoms and collected from Jimbour, Queensland, Australia, shared only 81% nt identity with its closest match, CpCV-C (JN989416).

**FIG 1:**
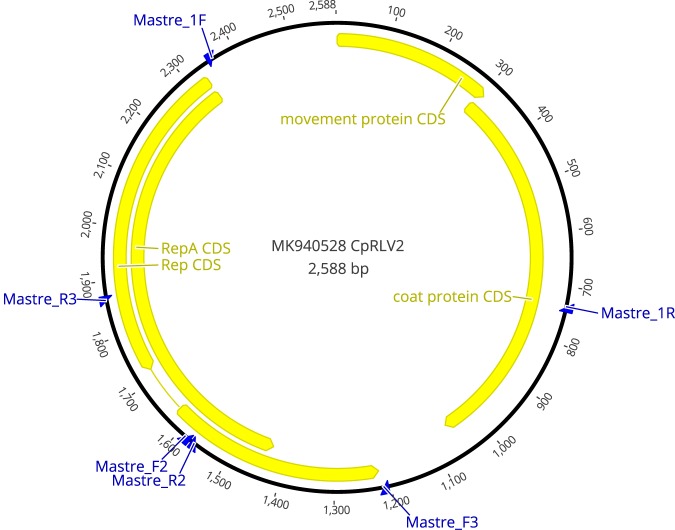
Annotated genome of chickpea redleaf virus 2 (CpRLV2), a new *Mastrevirus* species isolated from chickpea in Queensland, Australia. Blue arrows indicate the location of primers used for identification. Coding sequences (CDS) are represented as yellow arrows, pointing in the direction of translation.

The whole-genome sequence of isolate 5495 (Fig. 1) was obtained using the back-to-back primers Mastre_F2 (GANTTGGTCCRCAKATGWAGAG) (modified from references 1 and 6) and Mastre_R2, as well as Mastre_F3 and Mastre_R3 (SARTTYCCHCATGAATGGGC). The sequenced PCR products were assembled and analyzed (Geneious mapper and ClustalW, with default parameters) in Geneious 10.0.3 (Biomatters). The full sequence of isolate 5495, hereafter referred to as chickpea redleaf virus 2 (CpRLV2), has revealed a 2,588-nucleotide (nt) circular single-stranded DNA (ssDNA) genome with a GC content of 43% and an organization typical of mastreviruses. The closest match by BLAST (7) for the whole genome with 70% nt identity was CpCV-A isolate AU-2683A (GenBank accession number JN989413). According to the International Committee on Taxonomy of Viruses (ICTV), species demarcation for mastreviruses is less than 78% nt identity over the full length of the genome (https://talk.ictvonline.org/ictv-reports/ictv_online_report/ssdna-viruses/w/geminiviridae/394/genus-mastrevirus), indicating that CpRLV2 is a novel *Mastrevirus* species. At the amino acid level, the movement protein (MP) of CpRLV2, as well as the replication (Rep) proteins RepA and Rep, share 80%, 80% and 77% amino acid identity, respectively, with CpCV-A AU-2683A. However, the coat protein shares only 63% amino acid identity to CpCV-A AU-2683A, and the closest match with 75% amino acid identity was to chickpea chlorotic dwarf virus isolate Eco18 (GenBank accession number AM933135). Therefore, the main difference in the genome of CpRLV2 from that of CpCV-A AU-2683A, the closest relative, is the coat protein gene region.

### Data availability.

The sequence was deposited in GenBank with the accession number MK940528.
